# “REACH’’ for troubleshooting peripheral defocus myopia control spectacles

**DOI:** 10.3389/fopht.2025.1583599

**Published:** 2025-05-15

**Authors:** Sujit Shah, Gautam Motwani, Pavan K. Verkicharla

**Affiliations:** Infor Myopia Centre and Myopia Research Lab, Brien Holden Institute of Optometry and Vision Sciences, Prof. Brien Holden Eye Research Centre, L V Prasad Eye Institute, Hyderabad, India

**Keywords:** peripheral defocus spectacles, myopia control, counseling, education, troubleshooting

## Letter to the editor

The worldwide increasing trend in the prevalence of myopia and its associated complications has led to the development of various optical solutions for controlling myopia progression in the last decade (1).

With research highlighting the importance of counteracting peripheral hyperopic defocus (2), myopia control peripheral plus or defocus spectacle lenses have been made commercially available in multiple countries. These spectacles incorporate a central distance clear zone with plus power in the peripheral regions, creating a peripheral myopic defocus that is hypothesized to slow down the axial elongation. Although these lenses have demonstrated good treatment efficacy (2, 3), a certain proportion (albeit small) of children may experience or complain about visual disturbances with these spectacles despite achieving 20/20 central visual acuity for distance viewing at the time of prescribing. It is important that the quality of vision is not altered and there are no issues with the spectacles to achieve the best compliance and successful outcomes. However, the nature of the design of these spectacles is likely to influence visual performance in the initial days of lens wear. For example, a questionnaire-based study conducted in Chinese children demonstrated that the children had reduced mid-peripheral vision through the lenses compared with single-vision lenses (4).

**Figure 1:**
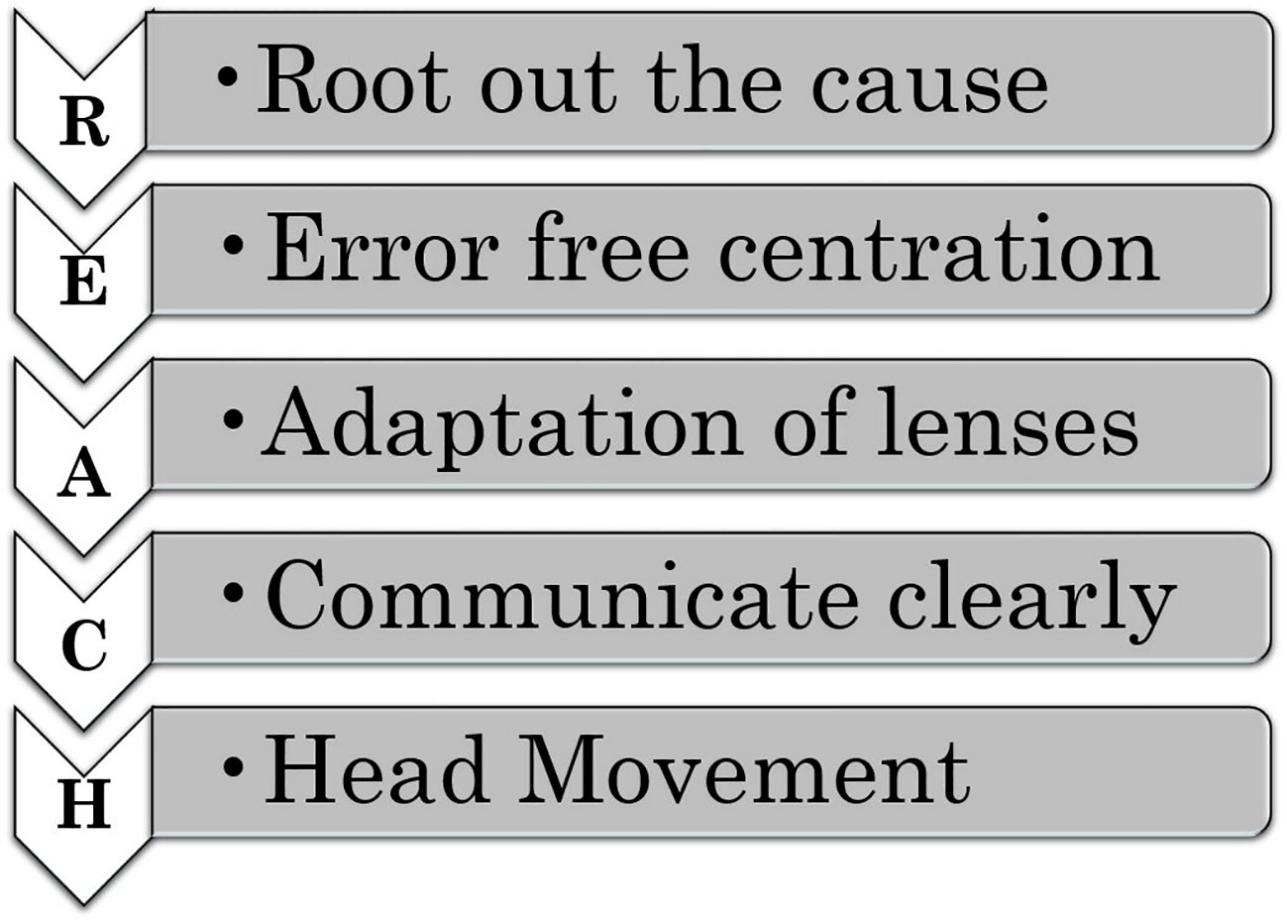
Five-step REACH guidelines for troubleshooting with myopia control spectacles.

These spectacles are relatively new to the ophthalmic clinical practice, and they present unique issues for the patients and practitioners that warrant the attention of stakeholders, especially for troubleshooting. Based on clinical experience in myopia management, we present a troubleshooting guide with the acronym “REACH” to address visual disturbances in children wearing peripheral defocus spectacles as indicated in **Figure 1**. This guide includes key aspects of patient-parent communication and strategies for managing concerns, illustrated through three real patient cases as indicated in **Table 1**. The insights a practitioner gets through REACH are critical to ensuring the success of myopia management, which can be seen below.

**Table 1:** Example cases where troubleshooting of peripheral defocus myopia control spectacles was conducted using the REACH guidelines.

REACH	Case 1: the corner-seated student	Case 2: the peripheral vision challenge	Case 3: the lens swap situation
R - Root cause	Blackboard blur due to classroom corner seating that forced the patient to look through defocus zones	Blur with slight eye movements; difficulty adapting to peripheral defocus design	Blurred vision and headache due to swapped lenses and a round frame affecting orientation
E - Error-free centration	Lens centration verified as correct; VA 20/20	Pupil alignment and lens positioning confirmed correct	Right and left lenses were swapped, causing centration issues.
A - Adaptation	Blur appeared after 20 days, requiring additional guidance on proper lens use.	The patient struggled with adaptation and needed extra support.	Adaptation was complicated by incorrect lens placement and sports-related challenges.
C - Communication	Explained seating-related defocus and proper viewing techniques	Educated the child and parents on lens design and peripheral defocus	Emphasized correct lens orientation and provided sports-specific guidance.
H - Head movement	Advised aligning with the board and trained proper head movement.	Taught head movement strategies for viewing peripheral objects.	Guided patient on proper head positioning after lens correction, especially for sports.

### R: Root out the cause

In clinical practice, it is crucial to understand the child’s concerns and experiences after wearing myopia control spectacles in spite of having 20/20 visual acuity on examination. Thus, one must root out the cause of the problem with these lenses by engaging in open-ended discussion-driven conversations to uncover the underlying problem that may impact vision and, in turn, the compliance and the effectiveness of myopia control treatment. Regular follow-up visits after initiating treatment are therefore important to monitor the child’s visual comfort, assess treatment progress, and address any concerns promptly. This proactive approach ensures that necessary adjustments are made in a timely manner, fostering optimal treatment success and long-term visual health.

### E: Error-free centration

It is essential to ensure that the clear central zone of the lens is properly aligned with the center of the pupil. Peripheral defocus spectacles from various companies have a central clear zone varying from 8 mm to 9.4 mm (2, 3, 5). Additionally, one should verify if there is any low riding of the frame on the nose such that the child is looking through the defocused zone, causing blurred vision. Larger frames with minimal pantoscopic tilt may help maintain the intended optical design and prevent unintended shifts in the defocus zone.

### A: Adaptation of lenses

Given that these peripheral defocus lenses are different from conventional single vision lenses, it is important to indicate the need for adaptation time with these lenses of at least 2–3 weeks. For children sensitive to peripheral blur, it is important to take additional care when introducing myopia control spectacles. One should highlight that the child needs ample time for them to adjust to the new visual experience, especially if they previously struggled to adapt to changes in single-vision spectacles or returned to the clinic soon after trying peripheral defocus spectacles.

### C: Communicate clearly

One should begin by explaining to both the child and their parents, in simple terms, how peripheral hyperopic blur works and its connection to myopia control spectacles. If possible, one should show them demonstration lenses and describe the design, nature, and purpose of these lenses, emphasizing how they help manage myopia and set the expectations upfront.

### H. Head movement

One should encourage the child to adopt a head movement strategy instead of relying solely on eye movements to view objects outside the central clear zone. By turning their head to direct their gaze, they can bring the objects into the clear central zone and minimize peripheral blur. Otherwise, the child is likely to indicate a perception of blurring when viewing objects in the peripheral visual field. This is especially relevant in school when looking at a whiteboard or LED screen while sitting in a corner or on one side of the classroom.

### Case 1

A 12-year-old with -3.50 DS refractive error in both eyes reported occasional blackboard blur after 20 days of wearing peripheral defocus spectacles. An investigation revealed the child’s habit of sitting in a corner of the classroom, which required him to look through the defocus zones and experience blur. As indicated in **Figure 1**, REACH we recommended a head movement strategy, being aligned with the board, and using head movements to view peripheral objects. In the subsequent follow-up, the child confirmed this adjustment resolved the issue completely.

### Case 2

A 11-year-old with refractive error (RE: -2.50DS/-1.00DC×180, LE: -3.00DS/-1.00DC×180) returned to our clinic a month after starting to wear peripheral defocus spectacles, reporting blurred vision with slight eye movements. On examination, visual acuity was 20/20 in both eyes, indicating good central vision. After confirming proper pupil alignment within the clear zone, we educated the child and parents on the lens design, stressing the need for adaptation and head movements to view peripheral objects. In the subsequent follow-up, the child reported complete resolution of the issue, demonstrating the value of counseling and support.

### Case 3

A 9-year-old with refractive error (BE: -4.00DS/-1.00DC×180 in both eyes) returned a month after being prescribed peripheral defocus spectacles, reporting blurred distance vision and frontal headache with no other ocular complaints. An examination showed a one-line reduction in vision despite the correct prescription. On further examination, the child was found to be wearing circular-shaped frames, and the issue was due to misaligned lens centration caused by swapping the right and left lenses in identical round frames. Adjusting the lenses resolved her symptoms.

Additionally, some children reported difficulties playing sports such as tennis and badminton with these spectacles, underscoring the importance of addressing individual needs and offering tailored guidance. Based on our experience, young adults who are likely to drive also expressed some level of discomfort with the defocus spectacles.

REACH can act as a guideline to prevent any complications or for troubleshooting, given that the complaints are multi-dimensional. It would be useful for a clinician to have a checklist in the form of a structured questionnaire to enhance the evaluation of the patient’s experience and discomfort.

## Conclusion

The REACH troubleshooting guidelines will benefit eye care practitioners when prescribing peripheral defocus spectacles for myopia control. These guidelines can help eye care practitioners to reduce multiple clinic visits, dropouts, and issues related to poor compliance when peripheral defocus spectacles are prescribed.
